# System-Level Scenarios for the Elucidation of T Cell-Mediated Germinal Center B Cell Differentiation

**DOI:** 10.3389/fimmu.2021.734282

**Published:** 2021-09-20

**Authors:** Niels J. M. Verstegen, Victor Ubels, Hans V. Westerhoff, S. Marieke van Ham, Matteo Barberis

**Affiliations:** ^1^Department of Immunopathology, Sanquin Research and Landsteiner Laboratory, Amsterdam University Medical Centers, University of Amsterdam, Amsterdam, Netherlands; ^2^Synthetic Systems Biology and Nuclear Organization, Swammerdam Institute for Life Sciences, University of Amsterdam, Amsterdam, Netherlands; ^3^Systems Biology, School of Biosciences and Medicine, Faculty of Health and Medical Sciences, University of Surrey, Guildford, United Kingdom; ^4^Centre for Mathematical and Computational Biology, CMCB, University of Surrey, Guildford, United Kingdom; ^5^Department of Molecular Cell Physiology, VU University Amsterdam, Amsterdam, Netherlands

**Keywords:** systems biology, T follicular helper cells, B cells, mathematical modeling, B cell recycling and differentiation, memory B cell, plasma cell, cytokines

## Abstract

Germinal center (GC) reactions are vital to the correct functioning of the adaptive immune system, through formation of high affinity, class switched antibodies. GCs are transient anatomical structures in secondary lymphoid organs where specific B cells, after recognition of antigen and with T cell help, undergo class switching. Subsequently, B cells cycle between zones of proliferation and somatic hypermutation and zones where renewed antigen acquisition and T cell help allows for selection of high affinity B cells (affinity maturation). Eventually GC B cells first differentiate into long-lived memory B cells (MBC) and finally into plasma cells (PC) that partially migrate to the bone marrow to encapsulate into long-lived survival niches. The regulation of GC reactions is a highly dynamically coordinated process that occurs between various cells and molecules that change in their signals. Here, we present a system-level perspective of T cell-mediated GC B cell differentiation, presenting and discussing the experimental and computational efforts on the regulation of the GCs. We aim to integrate Systems Biology with B cell biology, to advance elucidation of the regulation of high-affinity, class switched antibody formation, thus to shed light on the delicate functioning of the adaptive immune system. Specifically, we: i) review experimental findings of internal and external factors driving various GC dynamics, such as GC initiation, maturation and GCBC fate determination; ii) draw comparisons between experimental observations and mathematical modeling investigations; and iii) discuss and reflect on current strategies of modeling efforts, to elucidate B cell behavior during the GC tract. Finally, perspectives are specifically given on to the areas where a Systems Biology approach may be useful to predict novel GCBC-T cell interaction dynamics.

## 1 Introduction

Long lasting and effective humoral immunity depends on the generation of high-affinity, class switched memory B cells (MBC) and plasma cells (PC). Their differentiation occurs in germinal centers (GC), which are specialized structures that emerge in B cell follicles within secondary lymphoid organs after encounter of T cell-dependent antigen (Ag) (1). Each mature B cell expresses a transmembrane immunoglobulin, or antibody, which is also known as the B cell receptor (BCR). Immunoglobulins are composed of a heavy and a light chain that both contain a constant and a variable region. The immunoglobulin heavy chain constant region, also referred to as isotype, is classified in five main classes – the naive isotypes IgD and IgM and the class-switched isotypes IgG, IgA and IgE. Since these isotypes have different biochemical properties, the immunoglobulin isotypes also define the functional heterogeneity of these molecules (2–4). In the course of a humoral immune response, B cells adapt their isotype in a process called class-switch recombination (CSR) so as to ultimately produce immunoglobulins with the effector function most appropriate to clear the specific infection. The variable region of the BCR confers the Ag-binding site. During GC reactions, Ag-specific B cells are subject to somatic hypermutation (SHM), which induces random mutations in the Ag-recognition domains of the variable regions. This results in cells that express slightly modified BCRs and thereby exhibit an altered affinity for the target Ag (1, 5). GC B cells (GCBCs) that acquired an increased affinity for the target Ag are positively selected through interactions with the intact Ag retained by follicular dendritic cells (FDCs), and T follicular helper cells (Tfh cells) (6, 7). Repeated cycles of proliferation, hypermutation and repeated Ag capture and T cell-mediated selection eventually ensure formation of high-affinity B cells in the maturing GCs. The dynamic extracellular signals directing the GC cycles activate intracellular signaling networks within the specific B cells. Together with cell intrinsic properties, these signaling networks control B cell differentiation and determine whether a GCBC will resume proliferation and continue in the GC cycle or will leave the cycle in favor of terminal differentiation into either MBC or PC. Although great progress has been made in experimentally identifying the signals that steer GCBC fate determination, it remains challenging to study how the dynamic extracellular signals synergize, let alone how changes in signaling strength may affect the system. In view of the positive feedbacks in the GC cycle, and distributed control in cell signaling (8), it is to be expected that certain minor differences in signaling could make the difference between a proper and improper functioning of cellular immune networks (9), whereas others may be ineffective due to homeostatic mechanisms.

Elucidation of some signaling flows through this multicomponent, temporally evolving dynamic system may be possible experimentally. Strategic experimentation, designed and informed by earlier experiments and from biochemical, biophysical, cell biology and genetic knowledge, is sought. The tremendous amount of information involved then requires a systematic way of containing the information in a predictive way. Therefore, other inter-disciplinary fields of research may be of substantial help to capture the information flows within the intracellular networks in terms of integrating process activities and the regulation thereof. To address such dynamic processes, Systems Biology is on call to investigate how cellular functionality is achieved by integrating nonlinear molecular processes, employing computational, network-based approaches and detailed experimentation (10, 11). Although it will remain a challenge to comprehend the complex dynamics of the GC reactions considering the experimental scenarios available, integration of data coming from new, targeted experiments in an appropriated computational framework of the GCs, should enable progression to more robust and predictive understanding. Methodologies have been developed that enable to assess the relative importance of various processes and components in an intracellular network to predict network functions, as well as to assess how such processes are being regulated (12, 13). And, with the great advance of computing, an even larger variety of computational approaches has come about. In the present paper, we will examine Systems Biology approaches that integrate the concepts of regulation and mechanistic modeling in the context of quantitative experimental data. New with respect to immunology is this comprehensive systems biology angle. New to systems biology is the focus on the immunological phenomenon of GC maturation and the integration of intracellular and extracellular signaling.

We will examine which of these Systems Biology approaches and methodologies may be used to investigate intracellular networks that determine long lasting humoral immunity. Specifically, we will focus on the networks involving Ag and Tfh factors involved in GCBC fate determination. Therefore, we will i) identify intracellular networks that play a role in GCBCs fate determination, and ii) discuss how computational methodologies that have been developed may be integrated with current and new experimental scenarios. This will enable us to weigh the relative importance of Ag and Tfh factors, and hypothesize possible mechanistic explanations of the GCBCs differentiation process. This Systems Biology modeling has already lead to the elucidation of complex interconnected, highly non-linear networks or multiple redundant networks that regulate cellular differentiation, among which that of immune cells (14). In another example, this type of systems biology modelling has suggested ways out of the apparently irreversible transition from transient to chronic inflammation (15). In addition, these implementations of Systems Biology concepts may suggest missing factors or connections among factors. Prediction of functional networks and their regulators may be tested by design of additional, focused experimental strategies. Through iterative rounds of computational modeling and experimentation, hypotheses about functional mechanisms involved in the GC reactions may be predicted computationally and experimentally tested.

## 2 The Germinal Center Reaction

### 2.1 Initiation of a Germinal Center

**Figure 1:**
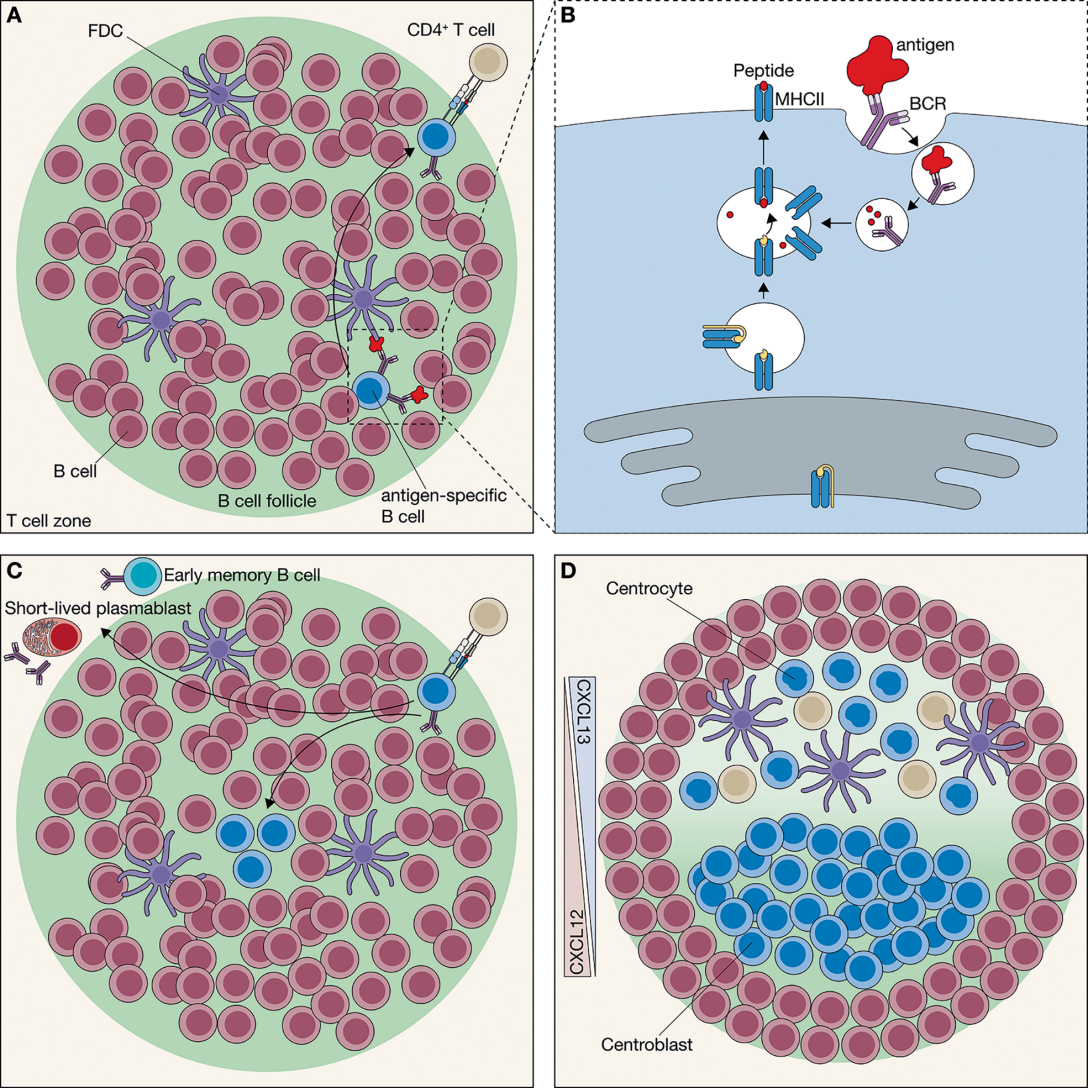
Initiation of the germinal center (GC) network. **(A)** In the secondary lymphoid organs, B cells are located in the B cell follicle. Follicular B cells engage with an Antigen (Ag) *via* their B cell receptor (BCR). Most Ag in the follicle is presented by follicular dendritic cells (FDCs), which retain the Ag for extended periods. Ag engagement partly activates the follicular B cells; however, to become fully activated, it requires interaction with a CD4^+^ T cells. Ag-engaged follicular B cells localize to the border of the follicle to encounter CD4^+^ T cells with the same Ag specificity. **(B)** To interact with the CD4^+^ T cells, the B cell needs to present Ag-derived peptide fragments through the major histocompatibility complex class II (MHCII). Thereto, Ag engagement results in BCR-mediated endocytosis, followed by Ag degradation and presentation of resulting peptide fragments through MHCII. **(C)** At the border of the follicle, CD4^+^ T cells screen many Ag-specific B cells to find the B cell with the same Ag specificity. At the time the CD4^+^ T cell encounters a follicular B cell with a corresponding Ag specificity, it provides the B cell with help that in turn results in the differentiation towards either short-lived plasmablasts, early memory B cells or GC precursor B cells. GC precursor B cells migrate towards the center of the follicle and starts hyperproliferation. **(D)** Hyperproliferation drives the formation of the mantel zone, which contains non-activated B cells. As the GC expands the chemokine gradient, mediated by CXCL12 and CXCL13, GC differentiation occurs into two phenotypically distinct zones, the dark zone (DZ) and the light zone (LZ). The CXCL12^+^ DZ is almost entirely populated by hyperproliferating centroblasts, whereas the CXCL13^+^ LZ contains FDCs, T follicular helper (Tfh) cells and centrocytes.

T cell-dependent B cell differentiation in response to so called thymus dependent Ags occurs in secondary lymphoid organs (SLOs). SLOs are populated by two lymphocyte populations – the B and T cells – that are largely segregated in two distinct areas through the action of multiple chemokine-receptor axes (16). Once B cells enter the secondary lymphoid organs, they migrate towards the CXC-chemokine ligand 13 (CXCL13) that is highly abundant in the B cell follicles. Instead, upon entry, T cells are directed to the surrounding T cell zone in response to the CC-chemokine ligands 19 (CCL19) and CCL21 (17). The follicles within the secondary lymphoid organs mostly include mature but Ag naive B cells, and are also populated by FDCs (**Figure 1A**). FDCs are non-migratory, long-lived stromal cells that derive from a mesenchymal precursor cell, and help to maintain primary follicles as B cell exclusive niches by secreting CXCL13 (18). Following passive influx or active transport of complement- and/or antibody-opsonized Ag into the follicle, the non-phagocytic FDCs bind and present the native Ag up to 12 months on the surface (19–23). Ag-specific B cells acquire intact Ag either by itself or after Ag-binding by FDC (**Figure 1A**) (18, 24–30). Engagement with Ag results in BCR-mediated signaling and Ag internalization. This is followed by intracellular degradation, and the generation of peptides that are presented on the B cells surface through major histocompatibility complex class II molecules (pMHCII) (**Figure 1B**). This presentation enables B:CD4^+^ T cell interactions (**Figure 1A**). Ag-engaged B and activated CD4^+^ T cells interact at the T-B border after directed localization from their separate zones. This localization is mediated through upregulation of CCR7 on the activated B cell (31) and through upregulation of CXCR5 and downregulation of CCR7 on the dendritic cell-activated CD4^+^ T cell (**Figure 1A**) (32). After cognate interaction *via* the pMHCII and the specific T cell receptor (TCR) on the B and T cell, respectively, the CD4^+^ T cells confers additional signals mediated through co-stimulatory molecules and cytokines that together determine the fate of the activated B cells (33). Co-stimulation through CD40 on B cells induces anti-apoptotic programs in the BCR-activated B cells and allows B cell survival and proliferation. In addition, the T cell signals may allow B cells to migrate to extrafollicular sites within the secondary lymphoid organs, where they differentiate into short-lived PCs to produce the first wave of antibodies exhibiting relatively low-affinity (**Figure 1C**) (34, 35). Alternatively, activated B cells acquire a GC-independent early MBC phenotype and enter the circulation (**Figure 1C**) (36–38). Finally, a limited number of B cells migrates back to the center of the B cell follicle after downregulation of CCR7 to start the GC reaction (**Figure 1C**) (1, 5, 39, 40). For these B cells, the CSR inducing them into IgG B cells was recently found to already be initiated during the initial B:T cell interaction prior to GC entry (41). Interestingly, whereas many individual naive B cells only produce one type of early effector cell, including short-lived plasma cells, GCBC and GC-independent MBCs, others were found to be able to take part in the various differentiation processes after initial B:T contact (42). This suggests that both internal stochastic and externally regulated processes facilitate activated B cell fate determination.

### 2.2 Maturation of the Germinal Center

**Figure 2:**
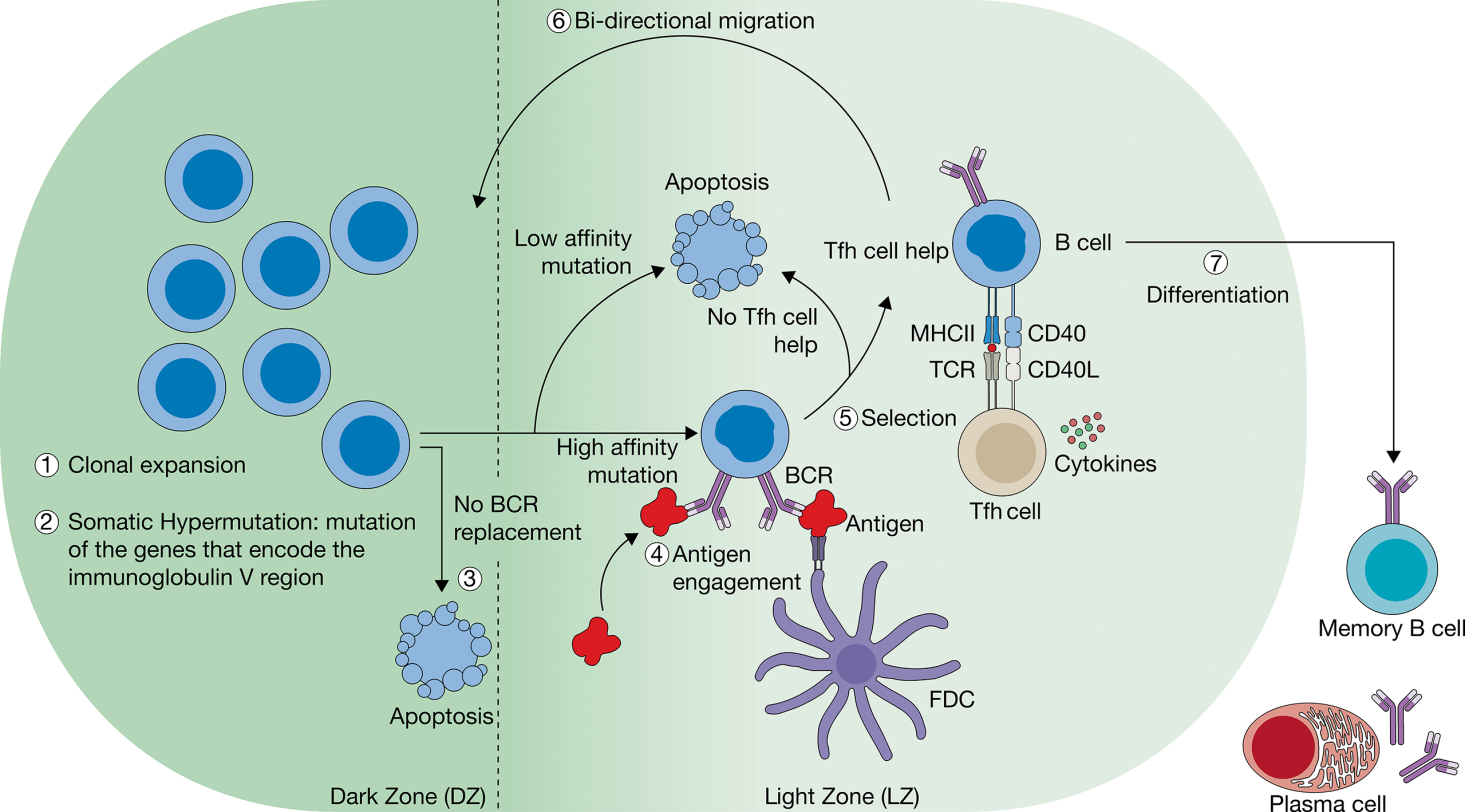
The germinal center (GC) reactions. In the dark zone (DZ) of the GC, B cells undergo clonal expansion (1), which is accompanied by spontaneous point mutations in the gene that encodes for the variable domain of the B cell receptor (BCR) (2). GC B cells (GCBCs) with mutations that compromise BCR expression are removed through apoptosis (3). GCBCs with functional BCRs test the potentially altered affinity in the GC LZ through interactions with the antigen (4) and Tfh cells, which provide CD40L and cytokines (5). GCBCs that obtained a higher affinity BCR are expected to outcompete those with weaker affinity as a result of these interactions (4 and 5). Ultimately, positively selected B cells are the only ones to survive and either re-circulate to the DZ (6) or differentiate into either memory B cells (MBCs) or plasma cells (PCs) (7).

It is estimated for certain Ags that three fully activated Ag-specific B cells clones reach the center of the follicle and start to proliferate (**Figure 1C**). The cell cycle time of GCBCs is estimated to be approximately 6-12 hours, making these cells rank amongst the fastest dividing mammalian cells (43, 44). Dividing GCBCs exhibit downregulated expression of the BCR, through which they are unreceptive to Ag during proliferation (45). The resident non-activated B cells that encircle the hyperproliferating GCBCs are pushed aside to form a mantel that surround the GC (**Figure 1D**) (1, 46). After initiation of an early GC, GCBCs continue to clonally expand in the absence of mutations to reach a population size of about 1,500 B cells around day 7 (46). Around the same time the GC start to polarize into two distinct zones, the dark zone (**DZ**) and the light zone (LZ), which were named as such based on their appearance using light microscopic analysis (**Figures 1D**, **2**) (47, 48). The DZ is almost completely populated by clonally expanding densely packed GCBCs that have a high nucleus-to-cytoplasm ratio, which gives this zone its “dark” appearance (**Figure 1D** and **Figure 2**). The GCBCs in the LZ are surrounded by FDCs and Tfh cells, through which this zone appears to be “lighter” (**Figures 1D**, **2**) (5). Hyperproliferating GCBCs, also called centroblasts (CBs), express the chemokine receptor CXCR4, which directs the migration towards the CXCL12-secreting reticular cells in the DZ (**Figures 1D**, **2**) (49, 50). The importance of CXCR4 in GC organization was shown in CXCR4-deficient mice, which exhibited disrupted GC polarization compared to wild type, as shown by the exclusion of CBs from the DZ (49).

GCBCs in the DZ are subject to the activity of activation-induced cytidine deaminase (AID), an intracellular enzyme that mediates the introduction of random mutations (somatic hypermutation or SHM) in the Ag-binding domains (i.e. complementarity determining regions) of the BCR (and later secreted antibodies) (**Figure 2**) (46, 51, 52). As GCBCs downregulate expression of their initial BCR prior to SHM, these reactions yield Ag-specific B cells with altered affinities for their Ag (53). CBs that acquired damaging mutations – mostly mutations that induce a frameshift or a stop codon – and fail to replace surface BCR are removed by apoptosis in the DZ (**Figure 2**) (53, 54). The CBs that post-SHM efficiently replace the BCR migrate to the LZ (**Figure 2**).

Migration to the LZ is initiated when CBs lose CXCR4 expression, which shifts the balance of chemokine receptor responsiveness in favor of CXCR5. It has been implied that this shift is regulated by a cell intrinsic “timer” or “counter” that controls the cellular localization of GCBCs (50). As a result of the shift in chemokine receptor responsiveness, CBs migrate towards the CXCR5 ligand, CXCL13, which is secreted by FDCs in the LZ (**Figure 1D**) (21). The now non-proliferating LZ GCBCs, or centrocytes (CC), are then subject to the selective pressure of Ag and Tfh cell help. CC that successfully maintained or enhanced their affinity in the GC DZ acquire Ag and are selected after cognate help from Tfh cells (**Figure 2**) (1, 45). Unless Ag is still present in excess, the CBs with lower Ag affinity will lose the competition with the cells with high affinity and succumb to apoptosis because they are not selected for survival. A CC that is selected for survival will either continue in the GC cycle and resume proliferation or will terminally differentiate into MBC or PC, which is regulated by the dynamic extracellular signals provided by antigen and Tfh cell interactions (**Figure 2**; see below for details). Alternating rounds of positive selection of high-affinity variants will progressively outcompete the CCs with low Ag affinity (**Figure 2**) (54). Eventually this process, coined affinity maturation, yields an Ag-specific B cell population with high affinity BCRs (43, 44, 54, 55), unless Ag remains present in excess.

Systems Biology principles may be expected to be relevant here for proposing hypotheses. First, a multitude of processes determines the outcome of CCs with high affinity receptors producing high affinity antibodies. Suspecting analogies with other complex networks, it is unlikely that any single one of the factors involved is the rate limiting step for the production of B cells. It is more likely that many factors will control the process simultaneously and that, depending on the Ag load, different factors will be more in control. Second, with respect to the ultimate affinity of the antibodies, it may be expected that some factors will exert either an activatory or inhibitory effect. Third, there will be a difference depending on whether antigenic determinants are offered on a single macromolecular (or cellular) structure. In case they are offered on the same structure, high affinity antibodies should be developed only against one of the antigenic determinants, whilst if they are offered on separate structures, they should be developed against all. This should be relevant if the infectious agent is able to mutate its antigens. Fourth, since proteins are the ultimately functional molecules, the control of their effectiveness should be expected to reside as much at the level of transcription as at the level of post-translational processing.

## 3 GCBC Selection: Recycling *vs.* Differentiation

### 3.1 Modeling the GCBC Recycling Process

Systems Biology helps to integrate information of different types in order to understand biological function. This does not imply that such activities were not already undertaken before Systems Biology took off as a discipline. In 1993 already Kepler and Perelson asked whether the proposed development of high affinity through mutation and selection in a single maturation step of B cells was consistent with established mutation rates. Their computational calculation determined that GCBCs should require a higher than physiologic mutation frequency during SHM if the affinities seen *in vivo* should be achieved through a single burst of mutations in the DZ (56). It was therefore suggested that, after LZ migration, GCBC should re-enter the GC DZ for further rounds of proliferation and SHM. Again typical for Systems Biology, the testing of this prediction required the development of a new experimentally methodology that allowed a prolonged tracking of GCBCs *in vivo* (57, 58). Indeed, the emergence of two-photon microscopic imaging that allows the prolonged imaging of GCBCs *in vivo* was required to confirm that GCBCs re-enter in a proliferative state for further rounds of SHM (55).

However, although early computational predictions of the recycling probability of CCs indicated a recycling rate between 70-85% (59), recent mathematical and experimental investigations revealed that this value had been overestimated and in reality lies between 25-30% (55, 60). The period during which CCs are recycled was mathematically predicted to last for no less than 42 hours, but not to exceed the 55-hour mark. During this time, all selected CCs were recycled at a variable rate and re-entered the phase of proliferation (59). High-affinity clones experienced a significantly lower recycling probability and a higher early GC exclusion rate as compared to their low-affinity counterparts (59, 61). A mathematical model has been proposed where positively selected GCBCs localize to the DZ as they retain the Ag-derived peptides, which distributed asymmetrically during division between the two daughter cells. Based on this model, the daughter cell that acquired the Ag-derived peptide complexes differentiates to PCs and leaves the GC, whereas its sister GCBC, which did not acquire Ag, proliferates and localizes to the LZ (60). This computational prediction that early emigration of high-affinity GCBCs is a deterministic process, beneficial for affinity maturation and the early immune response, is in contrast to the earlier hypotheses that deemed this process to be of a stochastic nature (62). But of course, stochastics is nothing but determinism by unidentified dynamic factors.

Numerous selection methods enabling GCBCs to re-enter or not the proliferative state have been propose by computer modeling throughout the past three decades, and almost as many have since been discarded by the scientific community as they are unable to reproduce experimentally verified aspects regarding general GC properties (45, 63, 64), efficiency of affinity maturation (65), robustness in FDC Ag-presenting site numbers (66, 67), or output of high affinity GCBCs (68). Therefore, only the hypotheses that are still currently pursued are presented in this section.

**Figure 3:**
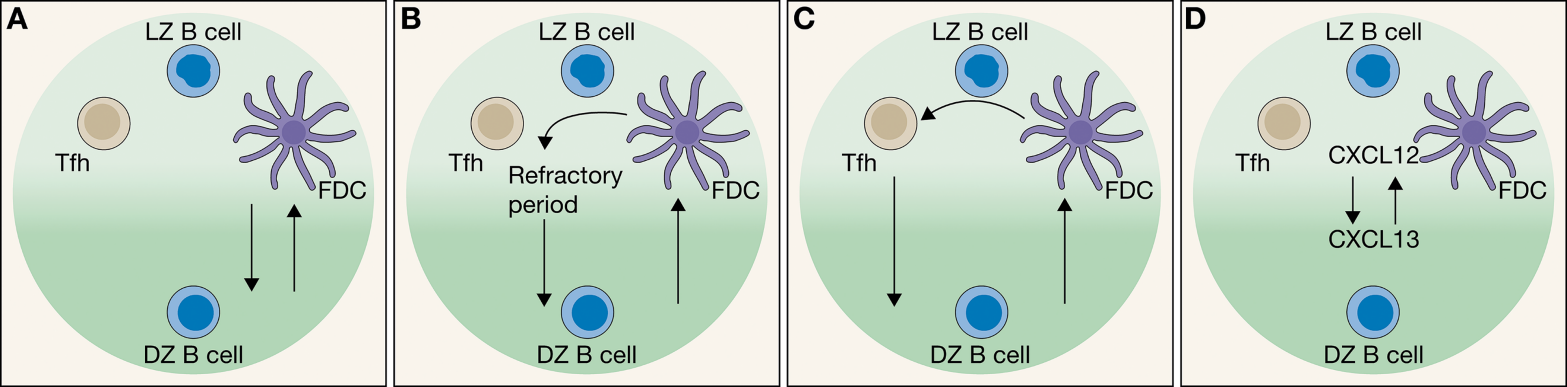
Models of Germinal Center B cell (GCBC) recycling between the light zone (LZ) and the dark zone (DZ). **(A)** Centrocyte (CC) selection through binding strength of the BCR to an antigen provided by follicular dendritic cells (FDCs). CC then migrates to the DZ and becomes centroblast (CB). Black arrows indicate the GCBC migration. **(B)** Increase in the refraction rate between CCs and FCDs. **(C)** Verification step, where CCs are selected by Tfh cells after a first selection by FDCs. **(D)** Spontaneous oscillations of GCBC between CXCL12 (representing the LZ) and CXCL13 (representing the DZ).

First, stochastic modeling of GCBC DZ re-entry suggests that a single first survival signal – where CC selection is determined by the binding strength of the BCR to Ag provided by FDCs – suffices to reproduce the rate of affinity maturation that it resembles an *in vivo* environment (**Figure 3A**) (69). In this scenario, the number of FDC sites on which Ag is presented is the limiting factor, as the variety of clones would compete for Ag binding for their survival and GCBC DZ re-entry. It is possible to envision that both Tfh and FDC interaction with GCBCs may induce forkhead box O1 (FOXO1) expression. As activation of the FOXO1–CXCR4 pathway leads to GCBC DZ re-entry and not apoptosis, this activation may be considered a pro-survival signal. Therefore, GCBC DZ re-entry can conceivably be primarily instigated not only by Ag interaction with FDCs, as previously thought (70, 71).

Second, an alternative method implements an increase in the refraction rate between CCs and FDCs (**Figure 3B**) (72, 73). In this hypothesis, a theoretical mechanism that occurs as a consequence of IgM-independent interactions between CCs and FDCs is introduced, where GCBCs that fail to bind to an FDC survive for a ‘refractory’ period of time after which they have an opportunity to bind to the FDC again. This hypothesis is based on early experimental findings that LFA-1/ICAM-1 and VLA-4/VCAM-1 mediate adhesion of GCBCs to FDCs (74). However, a recent discovery showed that Ags displayed on FDCs do not remain membrane bound permanently (75), but are instead rapidly internalized whilst remaining intact within a nondegradative compartment, and are then cycled back to the FDC membrane surface as intact antigens where they are able to select Ag-specific GCBCs (23, 75). As such, it may be envisioned that this process is the biological equivalent to the refractory period introduced *in silico*, with the specific time frame being the signal for apoptosis (72, 73). The phenomenon should enable FDC and CC to dissociate from one other after selection of the latter and to enable the latter to move away and make way for another, not yet assessed, GCBC. By limiting the total number of encounters between CC and FDC, higher-affinity clones will be favored, as their binding occurs at an earlier time as a consequence of the continued selection pressure (72).

Third, various models involve a validation by Tfh cells to ensure CC recycling to the DZ (**Figure 3C**). Initially, GCBCs must be selected by Ag retained by FDCs before a secondary interaction with Tfh cells would occur (72, 76, 77). As T cells constitute only the 5-10% of the GC cell population, this interaction may be highly competitive (60, 78). The interaction time between GCBC : Tfh cells correlate positively with DZ re-entry and the number of divisions (55, 79). The validation may even constitute a further selection: the Tfh cells are able to bind multiple CCs simultaneously, whereas only a single CC is polarized, i.e. the one with the highest binding affinity (80). Here the principle would be that dual consecutive selection (multiplying the probabilities) is more restrictive than additive selection. Furthermore, mathematical analyses predict that upon binding to the Tfh cell a high pMHCII density is vital to the GCBC divisions so that high MHC density on the membranes of the GCBC induces cell division whilst reducing the frequency of subsequent mutation (60). This suggests that GCBCs enter the S phase of the cell cycle when entering the LZ, but must reach the DZ prior to their entry into the G2/M phase (60). The mathematical models implement an additional control of CC and Tfh cells interaction, by introducing a predefined thresholds of minimal and maximal survival time that when exceeded induce apoptosis (72). Affinity maturation in these models is strongest when Tfh cell count is minimal and can rescue a given Ag-presenting CC more specifically as compared to a larger subgroup of Tfh cells (72); multiple Tfh cells should increase the probability of GCBCs with other than the highest affinity to survive and be selected. This finding is in line with an *in vivo* scenario where Tfh cells recognize only a small set of Ag epitopes as opposed to being susceptible to a broad range of Ag presentation (80). This methodology allows for the highest affinity maturation where 60-70% of all output cells have a high affinity. Furthermore, methodologies that rely on T cell help can be robust to large variations in Ag availability in the event that Ag acquisition and FDCs interaction is uncompetitive (72). This method has been tested both phenomenologically and including the impact of spatial cell distribution. Mathematical models that included the impact of spatial cell distribution did not find experimentally uncertain parameters that would change qualitative results (72). The model has been experimentally validated, but the results do not exclude other mechanisms (40, 55).

Finally, chemokine-driven receptor down-regulation is investigated as a potential alternative GCBC recycling mechanism, through a small sub-division of germinal center models (**Figure 3D**) (81). Gaussian distributions in which CXCL12 represent the LZ and CXCL13 the DZ is used to establish a simple chemokine field. The robust oscillatory behavior is observed especially when CXCR4 and CXCR5 receptors are reciprocally regulated (81), with a periodicity resembling the experimental observations. However, no indication is given about the extent to which such a mechanism contributes to GCBC recycling.

From the Systems Biology perspective, it is likely that various mechanisms play a role depending on the selection process of the antibodies. The fitness value of only generating antibodies with the highest affinity is limited, as it will take more time on average to reach the highest possible affinity and individual cells of the population may not have the ability to generate very high affinity antibodies. In some conditions it may then be better to develop antibodies with lower specificity more quickly, especially if the target is a rapidly expanding pathogen. The occurrence of both IgM and IgG supports this consideration that biological fitness is served by a hierarchy of mechanisms that differ in both temporal and spatial acuteness. The ability of some antigens to evolve (or be selected) rapidly within the infected individual would call for fast and less specific antibodies, and then only produced in a short period, thereby accepting cross reactivity with ‘self’. Dealing with tumor antigens, that are closer to ‘self’ would then require more selective and high affinity antibodies. This possibility may be relevant in the context of disease, where the frequency of tumor infiltrating B cells in the tumor microenvironment or in the tertiary lymphoid structures (TLS) is related to a positive outcome against cancer aggressiveness (82–86). And then there is the cost of the system, which is much higher for the multi-stage process leading to the antibodies with highest affinity. More diversified studies are needed that take into consideration this variegated function of the immune system.

### 3.2 Effector B Cells Arise From Germinal Centers

In addition to engaging in Tfh cell-mediated selection for further rounds of DZ proliferation and BCR/antibody diversification, GCBCs differentiate into either long-living MBCs or, eventually, antibody-secreting cells (44, 55, 87, 88). Two distinct categories of PCs can be found in the antibody-secreting cell compartment: short-lived proliferating PCs/plasmablast and non-proliferating PCs, which encapsulate into survival niches in the bone marrow where they can persist for decades to produce isotype-switched high-affinity antibodies (89, 90). In contrast, MBCs recirculate through the blood and the lymphoid organs and may provide a rapid response upon recognition of the same Ag (91–94). Human peripheral blood primarily contains distinct GC-dependent MBC populations, mostly of the unswitched IgM^+^ and the switched IgG^+^ MBCs (38, 95, 96). Similar to humans, immunization with a T cell-dependent Ag results in development of unswitched and switched MBCs in mice (97, 98). Upon re-recognition of the same Ag, switched MBCs differentiate into PCs, whereas unswitched MBCs induce proliferation and re-enter into a germinal center reaction (97, 98).

The MBC and PC output from a GC is determined by a temporal switch (99). Effector cells are generated in a sequential order, starting with unswitched MBCs, followed by switched MBCs and then by a delayed appearance of long-lived PCs. Differentiation into the MBC or PC compartment was shown to be linked to the Ag affinity of the BCR, where GCBCs with high affinity end up in the PC compartment and GC-derived MBCs are generally of lower affinity, where switched MBCs have a higher mutation load as compared to the unswitched MBCs (68, 95, 96, 100). Notably, GCBCs that receive signals in the LZ to undergo PC differentiation, first migrate back into the DZ to transit out of the GC *via* the DZ:T zone boundary (7, 60, 101). In contrast, MBCs leave the GC directly from the LZ. This suggests that the signals GCBCs receive to become PCs may resemble those that retain them in the GC.

Although the combined effort of computational prediction and experimental validation has been shown to be of substantial use to elucidate the GC reaction, the dynamic regulation that determines terminal GCBC fate determination remains to be elucidated. To model GCBC fate determination, the different stages that LZ CCs endure should be defined. *In vitro* data suggests B cell fate to be determined stochastically with probabilities arrived from an as of yet unclear signaling pathway, independently from cell-cell contact or asymmetric division (102). With the recent discovery that B cells divide asymmetrically three out of four times (103), this hypothesis becomes more appealing (104).

From the Systems Biology perspective, at ambient temperatures all actual processes are inherently stochastic. It is the relative magnitude of the dispersion and the nonlinearities of the processes involved that make this consequential. Because cell numbers (both FDC and Tfh) around the CCs are likely to be just a few at any point in time, and the cell proliferation processes are exponential and occur in bursts, if only due to the similarity of GC cycling time and cell cycle times at any point in space and time, the selection-process rates may readily vary by up to 100%, indeed predicting stochasticity to be actual. Fate determination starts with an extracellular signal, which is recognized and transmitted in the cell as a result of ligand-receptor interaction and subsequent intracellular signal transduction. For GCBCs, these extracellular signals are provided by Ag and Tfh cells. To understand how the integrated signals through these extracellular signals facilitate GC fate determination, insight into the transcriptional profiles corresponding to the three different fates – re-circulation to the DZ and differentiation into either MBC or PC – is relevant in order to identify and weight the interactions involved.

## 4 Transcription Factor Network Regulating GCBC Fate Determination

**Figure 4:**
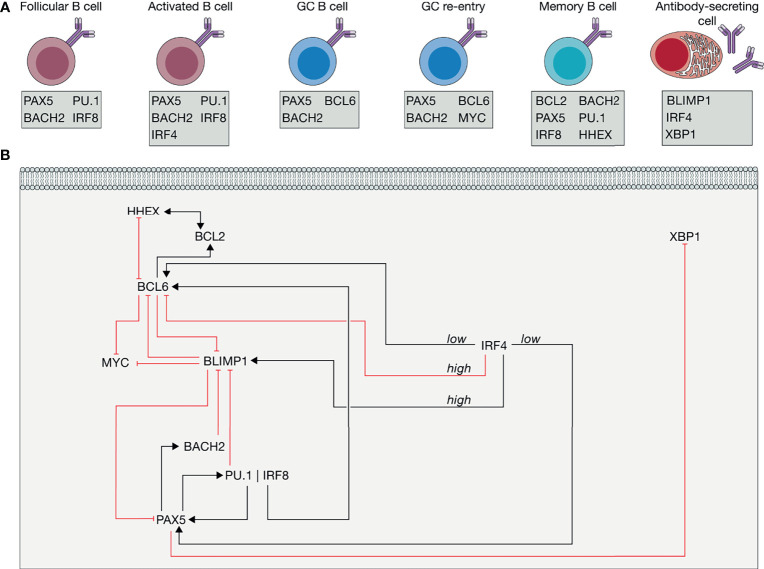
Transcriptional control of the germinal center (GC) and differentiation. **(A)** Transcriptional profile of follicular B cells, activated follicular B cells, GC B cells, GC B cells that re-enter into the GC reaction, high-affinity memory B cells (MBCs) and antibody secreting plasma B cells in terms of their phenotype-specific marker genes. **(B)** Transcriptional regulation of GC continuation and differentiation. Activations (black lines with an arrow head) and inhibitions (red lines with a flattened head) among genes and some proteins are shown. Relevant ones are: BTB and CNC homologue 2 (BACH2), B cell lymphoma 2 (BCL2), BCL6, B lymphocyte-induced maturation protein-1 (BLIMP1), Haematopoietically-expressed homeobox (HHEX), interferon-regulator factor 4 (IRF4), IRF8, Paired box protein 5 (PAX5) and X-box-binding protein 1 (XBP1) (see text for details).

**Figure 5:**
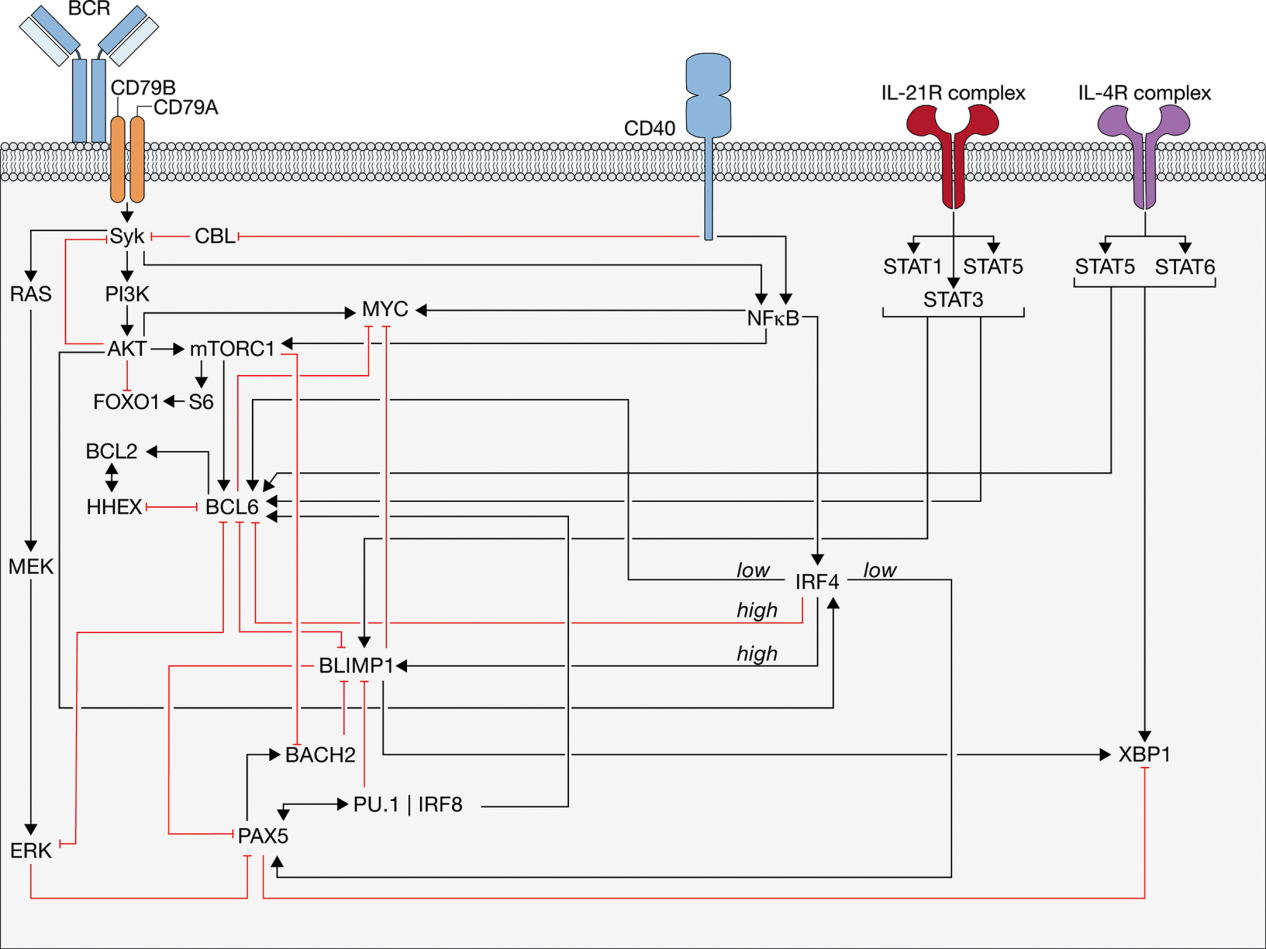
Control of the germinal center (GC) and differentiation mediated through receptor stimulation. The regulation of GC continuation and differentiation mediated through the different receptors-mediated signal transductions is shown. Activations (black lines with an arrow head) and inhibitions (red lines with a flattened head) among genes and some proteins are shown. Relevant ones are: BTB and CNC homologue 2 (BACH2), B cell lymphoma 2 (BCL2), BCL6, B cell receptor (BCR), B lymphocyte-induced maturation protein-1 (BLIMP1), cluster of differentiation 40 (CD40), Extracellular signal-regulated kinases (ERK), Forkhead box protein O1 (FOXO1), Haematopoietically-expressed homeobox (HHEX), Interleukins 4 receptor (IL-4R), IL-21R, Interferon-regulator factor 4 (IRF4), IRF8, mammalian target of rapamycin complex 1 (mTORC1), Paired box protein 5 (PAX5), Phosphoinositide 3-kinases (PI3K), signal transducer and activator of transcription (STAT) and X-box-binding protein 1 (XBP1) (see text for details).

GC initiation, continuation and GCBC fate determination are regulated by a large signal transduction network (see **Figure 5** below). The transduced signals regulate cell behavior, leading to the formation of cell phenotypes (identities) which serve dedicated functions (**Figure 4A**). The large network involves a complex and interconnected transcriptional network (**Figure 4B**). Follicular B cells express four transcription factors that put in place the signal transduction network responsible for their phenotype: paired box protein 5 (PAX5), BTB and CNC homologue 2 (BACH2), SPI-1 (also known as PU.1) and interferon-regulator factor 8 (IRF8). The latter two factors execute their function as heterodimer (105). PAX5 has been presented as the marker of B cell identity and positively regulates expression of BACH2, through which both factors are co-expressed throughout almost all mature B cell stages (106).

To differentiate into GCBC, naive B cells need to acquire expression of B cell lymphoma 6 (BCL6) (107). Upon activation, B cells start to express low levels of the transcription factor IRF4, which promotes GC fate through activation of BCL6 and PAX5 (89, 108, 109) (**Figure 4B**). PAX5 activates the IRF8 and PU.1 complex, which regulates GCBC development through the induction of BCL6 expression. The GCBC phenotype is preserved through transcriptional inhibition of the master regulator of PC differentiation, the B lymphocyte-induced maturation protein 1 (BLIMP1; also known as PRDM1) by BCL6 (110). To undergo further rounds of proliferation and SHM, the transcriptional profile of positively selected LZ GCBCs probably should not shift significantly from the GC profile stated above. Nevertheless, recent studies demonstrated that GC re-entry requires expression of MYC (111, 112), a cell cycle regulator that is transcriptionally suppressed by BCL6 (113, 114). Further analyses on the MYC-expressing GCBCs indicated that IRF4 expression was induced in these cells (111, 112). High levels of IRF4 transcriptionally suppress BCL6, whereas low levels could transcriptionally induce BCL6 (89), suggesting that expression of IRF4 may be sufficiently high to repress BCL6 and to allow expression of MYC required for GC DZ re-enter (**Figure 4B**). Since BLIMP1 determines PC fate, and repression of MYC is required but not sufficient for terminal differentiation, these data indicate that MYC may determine whether a GCBC re-enters the GC or starts terminal PC-differentiation.

Terminal differentiation into the PC fate starts with the upregulation of IRF4. In addition to its inhibitory effect on BCL6, high levels of IRF4 induce BLIMP1, which itself represses transcription of PAX5 and BCL6 (89). Furthermore, PCs express high levels of X-box-binding protein 1 (XBP1). XBP1 is an important regulator of immunoglobulin secretion, activated by BLIMP1 (115) and suppressed by PAX5 (89, 116–118). In summary, a network involving high expression of IRF4, its repression of BCL6 and therewith activations of BLIMP1 and MYC is crucial for transition from GCBC into PC.

The identification of the transcriptional program of MBCs is less apparent than for the GCBC and the PC. As compared to activated B cells, MBC exhibit enhanced expression of the pro-survival factor BCL2 (119, 120). To enable BCL2 expression, cells would need to extinguish BCL6 expression, since BCL6 suppresses BCL2 (121). Indeed, loss of BCL6 was shown to be the main driver of a pre-MBC transcriptional program and required for formation of human MBCs *in vitro* (122, 123). Recently, haematopoietically expressed homeobox (HHEX) was identified as a transcription factor regulating MBC differentiation through participation in downregulation of BCL6 (124). Similarly to GCBCs, MBCs show co-expression of PAX5 and BACH2, but also express PU.1–IRF8 (89, 125). Since the transcription factor complex PU.1–IRF8 negatively regulates PC differentiation, it is suggested that this complex may facilitate MBC formation (89, 126). However, no direct evidence supports this suggestion. Altogether, the aforementioned differences between the transcriptional profile facilitated by an Ag-mediated interaction and selection by Tfh cells are determinants of GCBC fate determination.

From the Systems Biology perspective, the signal transduction network (see **Figure 5** below) and its transcriptional sub-network involved (**Figure 4B**) are not just manifold but also complex. For example, BLIMP1 represses PAX5 which induces PU.1–IRF8 and thereby suppresses BLIMP1. This is just one example of the many instances of circular causation in the network: a factor may cause itself and thereby be its own effect. Moreover, BACH2 is both a cause and an effect of PAX5, just as much as PAX5 is both an effect and a cause of BACH2. In such cases, the analysis of changes in expression levels with variation in extracellular signaling or differentiation type, may lead to correlations that do not contain sufficient information to infer the complex cause-effect patterns within the complex network. To approach these issues, the network needs to be analyzed as a function of time after inducing well-defined perturbations within the network, bringing the results of the perturbations together in a single quantitative model of the network. Binary perturbations of the network, such as by homozygous knock out mutations are likely to invoke equally strong homeostatic responses of the biology around the network and thereby change network make-up unrecognizably. Such experiments should therefore be accompanied by verification that the structure of the network, i.e. the identity of the cell type, is not destroyed.

## 5 Regulation of GCBC Selection

### 5.1 Antigen-Mediated Receptor Stimulation

Following proliferation in the DZ and their localization to the LZ, the first signal that GCBCs receive is mediated by cross-linking of the re-expressed BCR by Ag. The different BCR-mediated processes – including Ag capture, peptide presentation efficiency and BCR signaling – should be included as a functional module within the comprehensive model of GCBC fate determination. There has been intense debate whether Ag-mediated BCR signaling directly mediates positive selection and differentiation of B cells or whether Ag-binding is just needed to progress to pMHCII expression and attract Tfh cell help and that only the latter controls B cell differentiation (68, 100). Ag capture is affected by the Ag concentration and the BCRs affinity. If the Ag concentration is too low, Ag-derived peptides are not presented by the GCBC and, consequently, will not receive help from Tfh cells; thus. the GCBCs initiate apoptosis and the GC collapses (127). In contrast, if the Ag concentration is too high, low affinity GCBCs will also be competent to be selected (127, 128). Also, GCBCs regulate their own fate through an antibody-mediated feedback mechanism. The high-affinity antibodies secreted by the PCs generated during the GC reaction are able to block target Ag bound to the FDCs (129). As such, non-differentiated GCBCs with the same specificity are not able to capture and present the specific Ag through which the epitope specific reaction is terminated. An Ag either freely migrates through the GC or associates with FDCs, which have diverse expression of receptors that hold the Ag (75). The specific binding epitope on the Ag is either not recognized or already bound by a GCBC that previously localized to the LZ. Therefore, the ability to capture a specific Ag is dependent on both the Ag concentration and the competition for binding by other GCBCs. The affinity threshold of B cell activation has a K_A_ of ca. 10^6^ – 10^10^ M^-1^ in the case of Ag bound to a membrane (130). In addition to the affinity for Ag, the overall B cell activation is affected by the BCR density. The combined functions of Ag affinity and BCR density is better known as Ag avidity.

Canonically, BCR stimulation above the avidity threshold results in the formation of a immunological synapse (maybe explain), which facilitates Ag processing and presentation (131). This synapse consists of BCR/Ag-FDC connection and associated protein micro-clusters, such as CD19 and LFA-1/ICAM-1 complexes (132, 133). Many assumptions regarding B cell synapse formation derive from extensive studies of the T helper cell synapse formation. However, differences can be observed regarding receptor densities, ligand affinities, mechanical characteristics, and extracellular length of receptor-ligand complexes. Contrarily to T helper cell synapse formation, the B cell synapse can still be formed when both the BCR and active transport processes are impaired and Ag acquisition is compromised due to undirected diffusion of synapse zone accumulating proteins (134). As such, any BCR modeling must contain a basal activation rate of the B cell synapse formation independent of Ag recognition (134). Stochastic Monte Carlo-based computational models have elucidated that, in addition to BCR/Ag driving protein micro-cluster formation, a progression in the affinity of LFA-1 may be necessary to induce synapse formation under membrane deformation circumstances (135). This implies that upon the binding of the BCR to Ag, signaling cascades should be initiated that direct LFA-1 to assume a conformation with a higher affinity binding for ICAM-1 (135). However, when BCR affinity exceeds a K_A_ value of 10^8^ M^-1^, this shift in LFA-1 affinity no longer suffices to induce synapse formation, indicating that still unknown synapse formation mechanisms must exist for high BCR affinity and membrane deformation (135). The LFA-1 conformation change should lead to membrane deformation accommodating BCR/Ag complexes at their equilibrium bond length and, subsequently, the B cell synapse (135). This should then allow for synapse formation across the range of physiological BCR affinities.

Furthermore, detailed modeling efforts show that relocation of BCR/Ag complexes to the B synapse is likely facilitated by cytoskeleton-mediated transport (136). Mathematical models show that directed transport of BCR/Ag complexes to the center of the synapse is capable of forming canonical synapse patterns should mechanisms based on differences in bond properties between the B cell synapse protein complexes fail to do so (136). Cytoskeletal-mediated transport of B synapse formation contradicts with the mechanism of T cells’ synapse formation. In T cells, the difference in the equilibrium bond length of TCR/MHCII and LFA-1/ICAM-1 is sufficient to induce segregation in the immunological synapse pattern (137, 138). Whether this bond length difference-mediated synapse formation can be accomplished solely by diffusion or relies on activate transport of receptors remains unclear (135). Regardless, as the equilibrium bond length is identical between BCR/Ag and LFA-1/ICAM-1, in the B cell spontaneous segregation into the synapse formation is unlikely to occur (136, 139).

Canonically, BCRs are cross-linked with Ags during the synapse formation, facilitating BCR-mediated signaling (132). Indeed, selected CCs exhibit active BCR receptor signaling. However, in these cells, the signaling is markedly reduced as compared to activated B cells (140). Attenuated proximal BCR signaling is mediated by hyper-activated phosphatases (141, 142) and high expression of the E3 ubiquitin ligase Casitas B-lineage lymphoma (CBL), which, among others, tags a mediator of BCR signaling, Syk, for degradation through its ubiquitylation (**Figure 5**) (143, 144). BCR ligation in GCBC results in a transient activation of Syk that rapidly decays (145). The rapid pulse of pSyk induces activation of the phosphoinositide 3-kinase (PI3K)-AKT pathway, which creates multiple negative feedback loops and dampens proximal BCR signaling in GCBC (**Figure 5**) (142). Nevertheless, transient phosphorylation of AKT allows for an efficient inactivation of FOXO1 (145), which controls GCBC proliferation and, by controlling the expression of CXCR4, allows DZ localization (146–148). Considering that BCR signaling is directly linked to FOXO1 degradation, these data together indicate that DZ re-entry requires only limited BCR ligation to allow FOXO1 activity.

The cross-linking of the BCR increases the rate of internalization, whereas the intracellular trafficking to the MHCII loading compartments is not affected by the cross-link (149). It was observed that the Ag presentation efficiency of B cells is dependent on the affinity of B cells for the Ag (150). An Ag that binds the BCR with high affinity remains associated during the trafficking to the MHCII loading compartment, through which peptide presentation by high affinity GCBCs is more efficient as compared to low affinity GCBCs. As such, high affinity GCBCs internalize and present more pMHCII. The density of peptide presentation potentiates the recognition of the GCBCs by Tfh cells with the same Ag specificity. pMHCII can vary in length from 11-30 amino acids in length (151). The internalized Ag is degraded intracellularly into a large number of possible peptides that may be loaded in the peptide binding pocket of MHCII and subsequently presented. Peptides with the highest binding affinity for the given MHCII allele will be preferentially expressed due to the peptide editing actions of HLA-DM and HLA-DO (152–155). This whole process mediates binding of cognate Tfh cells through the peptide-specific TCR to the Ag-activated GCBC. The BCR signaling output is altered by extending the time over which the BCR is stimulated; as a consequence, GCBCs that have a prolonged interaction with an Ag obtain significantly higher activity of intracellular signaling transduction. In summary, Ag recognition does not only provide the GCBC with the first intracellular signal transduction that potentially prepares the cell for differentiation, but it also promotes the interaction with Tfh cells.

From the Systems Biology perspective, B cell activation may well cause bursts of transcription, through its dependence on the activation of multiple BCRs, and of transcriptional silencing, due to the intracellular cycling of the BCR antigen complexes. The functional consequence of this decision making results in the cell exhibiting a phenotype switching to specific states of differentiation.

### 5.2 Tfh Cells Orchestrate GCBC Fate Determination

Before the discovery of the role of Tfh cells in the GC reactions, it was generally thought that only competition for Ag was responsible for GCBC selection. Nevertheless, it has been mathematically predicted, and recently experimentally validated, that selection of GCBCs is highly driven by competition for the pro-survival and mitogenic signals provided by the Tfh cells (40, 55, 72, 77). Tfh cells are specialized CD4^+^ T cells, which differentiate from naive CD4^+^ T cells after being primed by Ag-presenting cells (APCs), such as dendritic cells (DCs) and stabilized for the Tfh phenotype by cognate B cell interaction during Tfh priming (156, 157). Tfh cell differentiation is orchestrated by IL-6 and IL-21 in mice (158), and by IL-21, IL-12, IL-23 and TGF-β in human (158, 159). The interplay between these signals promotes induction of the Tfh cell phenotype through expression of the Tfh-determining transcription factor BCL6, while simultaneously inhibiting differentiation into the canonical CD4^+^ T cell subtypes, Th1, Th2 and Th17 cells (160). Phenotypic hallmarks of Tfh cells are high expression of CXCR5 and CXCR4 and low expression of CCR7, important for their correct localization to the GC LZ (161). In addition to Tfh, T follicular regulatory (**Tfr**) cells populate the GCs (162, 163). Because Tfrs seem mainly involved in termination of GC responses, they fall beyond the scope of this paper.

Tfh cells migrate through the LZ and constantly screen surrounding GCBCs through short-lived interactions in search for cognate pMHCII. Tfh cells are able to distinguish the high-affinity from the low-affinity CCs. The selection of high-affinity CCs is likely mediated by the number of pMHCII they present; in fact, high-affinity interactions between the BCR and specific Ag results in a higher amount of peptide presentation as compared to low-affinity interactions. This implies that Tfh cells are somehow capable to count pMHCII. Information on the amount of pMHCII on the CCs is likely provided through mechanical forces, which is also used by GCBCs to discriminate Ag affinity (164). They do this through pulling-forces that mediate rupture of single low-affinity bounds, whereas high-affinity, multivalent BCR clusters remain connected. Such a mechanism could also be used by the Tfh cells to provide help to high-affinity GCBCs. Non-cognate B and Tfh cells show an average motility of 6.6 and 9 μm/min, respectively (43, 44, 79). Upon cognate TCR/pMHCII recognition, a stable synapse between GCBC and Tfh cell is formed, decreasing their average motility to 4.16 μm/min (165). The stable synapse formation now allows the additional Tfh signals needed to support further GCBC differentiation such as co-stimulation and cytokine secretion.

#### 5.2.1 CD40 Co-Stimulation

An important interaction between GCBC and Tfh cell is mediated by the binding of CD40 to CD40 ligand (CD40L) (**Figure 2**). The CD40-CD40L pair is concentrated in the immunological synapse after cognate TCR/pMHCII recognition (166). The importance of CD40-CD40L interaction in humoral responses to T cell-dependent antigens was identified in patients with a congenital CD40L deficiency that causes X-linked hyper-IgM syndrome (167–171). These patients are unable to undergo isotype switching *in vitro* and *in vivo.* In agreement with these findings, mice with targeted disruption of CD40 or CD40L genes exhibit a similar phenotype (172, 173). Since patients and mice carry non-functional CD40L on all CD4^+^ T cells, it is not possible to determine which among the interactions at the B/T border or within the GC LZ is most critical. Administration of a CD40L blocking agent during the early phase of a T cell-dependent immune response abolished completely GC formation and reduced drastically serum antibodies, whereas its administration in the late phase almost completely dissolved the established GCs (174). Furthermore, late administration of the blocking agent decreased the affinity of the antibodies secreted by the PCs, as well as the amount of Ag-specific MBCs (175). These observations indicate that CD40L is required for the induction and continuation of the GC cycling, likely as a result of the incapability to inhibit the pro-apoptotic signals that GCBCs receive upon BCR ligation without being rescued by CD40 signaling. Recently it was demonstrated that interaction between the surface proteins Inducible T-cell co-stimulator (ICOS) and ICOS ligand (ICOSL) on the Tfh cells and GCBC, respectively, augmented CD40 signaling and promoted CD40L expression on Tfh cells; in turn, CD40-mediated signaling induced the up-regulation of ICOSL expression on the GCBCs (176). As such, ICOS and CD40 together facilitate an intercellular positive feedback loop that promotes GCBC-Tfh cell contacts.

CD40L-mediated clustering of CD40 promotes recruitment of tumor necrosis factor receptor (TNFR)-associated factor (TRAF) adaptor proteins (177, 178), which promote the activation of canonical and non-canonical NFκB pathways (179, 180) and facilitate activation of mitogen-activated protein kinase (MAPK) and PI3K (**Figure 5**) (145, 181–189). Recently it was determined that attenuated proximal BCR signaling can be abolished by CD40-mediated degradation of CBL (**Figure 5**) (143). Removal of CBL should abolish the degradation of Syk, boosting BCR signaling intensity to the PI3K/AKT and extracellular signal-regulated kinases (ERK) pathways. Herewith, BCR and CD40 signaling synergistically induce expression of MYC and IRF4, and activate the AKT-mediated mammalian target of rapamycin complex 1 (mTORC1) pathway, which facilitates phosphorylation of the ribosomal protein S6 (**Figure 5**) (7, 112, 145, 190). pS6 subsequently induces expression CXCR4 *via* FOXO1 to promote DZ re-entry, whereas MYC promotes the cell cycle progression. Active ERK inactivates BCL6 and PAX5, by rapidly degrading BCL6 though the ubiquitin/proteasome pathway and by repressing PAX5 activity (**Figure 5**) (191, 192). Loss of functionally active BCL6 and PAX5 results in expression of BLIMP1, which in turn protects its transcriptionally active state through suppression of BCL6 and PAX5 (89). Altogether, these observations indicate that BCR signaling and CD40 ligation are important for GCBC fate determination, notwithstanding additional signals provided by Tfh cell cytokines further supporting GC reactions.

#### 5.2.2 Cytokine Secretion by Tfh Cells

IL-21 and IL-4 are the two main cytokines secreted by Tfh cells and promote the fate determination of positively selected GCBCs (193). Tfh cells progressively differentiate along with the GC cycling to fine tune humoral immunity (194, 195). During the initiation of a GC, the first Tfh cells to emerge express IL-21. As the GC response progresses, Tfh cells gradually switch from IL-21 to IL-4 production. Interestingly, although the cytokines could be expressed simultaneously, secretion is limited to either IL-21 or IL-4. Within the GC, IL-21 Tfh cells are located on average more proximal to the DZ as compared to IL-4 Tfh cells (194). The cell-surface expression density of CD40L is significantly higher in Tfh cells that express IL-4 than in those that express IL-21 only (194). All of these differential characteristics suggest that cytokine-expressing GC Tfh cells have different roles in the GC.

IL-21 exerts its biological activities through interaction with the IL-21 receptor (IL-21R), whereas IL-4 can interact with two cell surface receptor complexes – the Type I IL-4 receptor and the Type II IL-4 receptor, which are both expressed by GCBCs. Receptor stimulation results in activation of Janus kinase–signal transducer and activator of transcription (STAT) signaling pathways (196). IL-21R signaling is predominantly mediated by STAT3, STAT1 and STAT5 (**Figure 5**) (197, 198), whereas signaling through IL-4R is facilitated through STAT5 and STAT6 (**Figure 5**) (199, 200).

In response to IL-4, GCBCs undergo a more pronounced isotype switching to IgG1 and differentiation into plasma cell, which may partly result from the enhanced CD40L expression in these different subsets (194). In contrast, IL-21 Tfh cells exhibit an increased rate of high-affinity mutations (194). IL-21 signaling was shown to play a large role in instructing the CC to CB transition (195), which is essential for the iterative rounds of SHM and affinity maturation. IL-21 induces the expression of both BLIMP1 (197, 201) and BCL6 (**Figure 5**) (201–206). The balance between induction of either BCL6 or BLIMP1 may be mediated through the STAT signal transduction; STAT3 is important for the IL-21R-mediated induction of BLIMP1 expression (197), whereas both STAT3 and STAT1 induce BCL6 (207). Alternatively, the signals that GCBC receive prior to IL-21, such as BCR and CD40 signaling, could make them more prone to induce either BCL6 or BLIMP1. Considering that BCL6 and BLIMP1 antagonize each other’s expression to help decide between GCBC and plasma cell differentiation, respectively, it is likely that, once a specific trait has initiated, IL-21 signaling is able to maintain it. This process may be observed early in the T-dependent Ag response. Here, high affinity B cells are predominantly observed in the extrafollicular plasma cells response, whereas clones with weaker Ag reactivity were primarily directed to GC reactions (35). As described above, prominent BCR and CD40 signaling responses induce high levels of IRF4 to enhance BLIMP1 and inhibit BCL6 expression. In this scenario, additional IL-21 signaling would synergize to complement the Ag-driven induction of BLIMP1. Conversely, weak BCR and CD40 signaling results in low IRF4 expression to enhance BCL6 and PAX5 expression, which both suppress BLIMP1 expression. In this way, IL-21 signaling would support BCL6 expression to promote GC retention, as observed (195), until the transcriptional program shifts due to affinity maturation to then promote plasma cell differentiation.

The observation that GC Tfh cells, similar to GCBCs, mature phenotypically and transcriptionally throughout the GC response suggests a reciprocal relationship between GCBC and Tfh cells. Their contacts elicit transient and sustained increases in intracellular free calcium in Tfh cell that is associated to multi-functional Tfh cells and is driven by TCR/pMHCII interactions (165, 176). Ag dose and BCR affinity, which change over time, affect TCR signaling strength and duration through the number of Ag-derived pMHCII molecules displayed by Ag-presenting B cells (28, 208–210). The amount of TCR signaling can have qualitative effects on CD4^+^ T cell differentiation into the specialized effector cell lineage (211), and may change the cytokines that are expressed by a specific lineage, such as IL-21 and IL-4 in Tfh cells, to achieve the required output response.

From the Systems Biology perspective, the interplay among the cytokines secreted by Tfh cells to promote GCBCs fate determination may well be non-linear, similarly to the regulations occurring in the GC signal transduction network (**Figure 5**) and its transcriptional sub-network (**Figure 4B**). Among the cytokines, IL-21 and IL-4 appears to play a major role in B cell differentiation, and a strategy that integrates predictive modeling to quantitative experimentation may point to the non-linear regulations occurring among the diverse cytokines involved in the process.

## 6 Discussion

The molecular cross-talk provided through Ag and the physical, biochemical and expression-mediated interactions between GCBCs and Tfh cells in the GC LZ orchestrate GCBC fate determination. Great efforts have been made to characterize the complex intracellular signal transduction and gene expression networks that mediate these interactions. Characterization and integration of the cellular signaling pathways should help to elucidate how the extracellular signals transmitted through these interactions lead to GCBC re-circulation or differentiation. Nevertheless, since the system is coordinately regulated by multiple receptors which impinge on specific transcription factors involved in fate determination, it has been troublesome to comprehend the complexity of the GC reactions considering the available experimental scenarios that often focus on the analysis of the effects of one or a few components in a more or less static ‘on-off’ approach.

This is now changing with the development of sophisticated experimental methodologies such as single cell sequencing, in principle able to deliver a map of transcriptomes over the GC. With single cell analyses of the important regulators, this may soon advance to the protein level. But we are not quite there yet, as the massive data flows and detailed manipulation experiments with spatial resolution at the scale of the GC are still a matter of the, near, future. The shift to data driven biology is challenging, as the number of experiments needed in a completely data driven approach would be vast. For example, measuring all the *in vivo* parameters driving B cell development into different phenotypes. Where bioinformatics aids in clustering the data according to defined criteria, dynamic methodologies are needed to simulate the implications of the data we obtain for the data we cannot obtain. This challenge then requires integration of predictive modelling with precise experimentation, what we have indicated here as Systems Biology. The application of principles developed and discovered in simpler networks could be of relevance for the understanding of how the GC works. Thus, modeling of its regulatory system may predict and elucidate how the fate of a GCBC is determined, followed by dedicated experimental testing.

One way to model an intracellular regulatory network is through a directed graph (212), which visualizes the complex regulatory network that selects positively GCBCs. Such a graph can be translated into mathematical equations that, when simulated, may lead to the identification of temporal mechanisms of GCBC differentiation. We have recently illustrated this for the network underlying cell cycle control and reactive oxygen species (ROS) production where we discovered new regulatory patterns (213, 214). The intracellular regulatory network involved in the positive selection of GCBCs should be modeled similarly as a multi-component, temporally evolving dynamic system. For such a model, sets of differential equations are applicable, which may include time and/or space dependent variables (212, 215), where space may be divided into a limited number of representative compartments with transport in between.

Differential equations are divided into two main groups, the ordinary differential equations (ODEs) and the partial differential equations (PDEs) (212, 215), whilst for each there are deterministic and stochastic types. ODEs are widely used and well-studied to analyze genetic, signal transduction and metabolic networks, by using concentration of components as a time-dependent variable. Nevertheless, in eukaryotes, cellular components rarely function in one single compartment; they rather shuttle between different compartments, and ODE equations may then be used to treat the dynamics of cellular components in different compartments separately, with additional rate equations for the transport processes between the compartments. When concentration gradients may exist within compartments, then the use of PDEs may be considered. In a PDE, a concentration is not only dependent on time, but also on three continuous space-dependent coordinates. As such, a PDE equation considers that molecules may behave differently in different areas of any same compartment. Although a PDE-based model more closely resembles biological realism, it requires increasing computing software as compared to ODEs, but worse, it requires more molecular information that is not always available. The use of an ODE-based over a PDE-based approach should be considered on a case-by-case basis. For the intracellular aspects of the GC, ODEs may well be suitable, as it has been recently shown in a computational model that recapitulated the switch from memory B cell to PC generation during the course of the GC reaction (216).

One of the relevant questions to be addressed regards the affinity, thus the quality, of different types of antibodies. The affinity-dependent selection serves the dual purpose of dealing with multiple novel antigenic determinants at relatively high concentration early on, and of subsequently removing the last few copies of more defined antigen. Affinity between macromolecules is to a significant extent determined by the number of water molecules they exclude upon binding as well as by the surface area through which they interact energetically, and thereby by the extent to which their binding surfaces are complementary in shape and able to squeeze out all the water molecules between them. Early models (59, 217, 218) thereto developed the concept of shape space as a way to comprehend Ag-antibody affinity. This complementary principle uses Ags to define where the antibody of maximum affinity is positioned in the shape space. This distance of the antibody to the theoretical optical clone is defined in terms of the minimum number of mutations necessary for the shape of the optimal clone to be reached, as a measurement of antibody affinity to the Ag. Affinity is subsequently estimated using a Gaussian function with this distance as argument, the distribution being mapped by characterizing a number of known mutations. This principle fails to predict all the experimental information, thereby highlighting the inherent unreliability of using shape space alone to define antibody affinity (72). In addition to shape, a network of other interactions dependent on electric charge, polarity, and hydrogen bridge formation of amino acid residues, will play a role.

Large scale systematic experimental data sets can be integrated into appropriate computational frameworks, to weigh the relative strengths and dynamics of the different signaling networks that control GC dynamics. To exemplify the necessity for systems-level approaches to the adaptive immune system, the interaction between Tfh cells, cytokines, and GCBCs to propagate the maturation process may be further investigated by training neural (artificial intelligence) networks on the data sets. This may generate a model that is able to predict the effects of changes in parameters. In addition, the neural network obtained may not heed laws of physics, chemistry or biology. The future is represented by neural networks trained on the experimental data sets as well as on artificial datasets produced by networks constructed on the basis of established physics, chemistry and biology.

Although, canonically, Tfh cells are presumed to have their lineage traced to Th1 and Th2 branches, recent studies show Tfh cells to have a distinct gene expression profile unlike Th1, Th2, Th17 or Treg cells (219). As cytokines have been confirmed to promote both T and B cell differentiation, understanding cytokine dynamics is vital, also when examining possible bistabilities of the |GC cycle, as it occurs in other immunology cycles (9). Although details of the specific mechanisms through which these dynamics occur are beyond the scope of this paper, a few considerations may be the starting point of future research. Secretion of IL-21 and IL-4 has been confirmed, but the influence that other cytokines have on Tfh cells, apart from germinal center formation, is largely unexplored. Also, the reciprocal relationship between GCBC and Tfh cell is of substantial interest. GCBCs may influence their own fate *via* the amount of pMHCII that determines the cytokines that are secreted by Tfh cells. Limited qualitative work has been done to elucidate the effects that cytokines have on T and B cell differentiation, generally neglecting the influence of temporal factors and of T cells secreting uncommon or multi-phenotypical cytokine profiles. Furthermore, recent investigation have highlighted that the effectiveness of T cell functioning is qualitatively, quantitatively and temporally determined by cellular and humoral signals such as cytokines (220). Anticipating the need for a system-level analysis to dissect the mutual regulation between GCBC and Tfh cells, these scenarios may be investigated through detailed kinetic models that consider the regulatory, activatory and inhibitory interactions occurring among the various signals involved. Simulation of these computer models will then identify critical network components, as well as mechanistic explanations for both the T and B cell differentiation processes specifically through cytokines-mediated regulation. Modeling of this system may enable identification of the components that are most prone to biological intervention (221). It may also suggest improved vaccination strategies and therapeutic agents that can be employed for the treatment of B cell-mediated malignancy or auto-immunity.

## Author Contributions

MB and SH conceived the study. MB and NJMV designed the study. NJMV, VU, and MB wrote the manuscript, with contribution from SH and HW. MB and SH provided scientific leadership and supervised the study. All authors contributed to the article and approved the submitted version.

## Funding

This work was supported by the Systems Biology Grant of the University of Surrey to MB, and by the EU ERASMUS+ Traineeship Grant to MB (recipient VU).

## Conflict of Interest

The authors declare that the research was conducted in the absence of any commercial or financial relationships that could be construed as a potential conflict of interest.

## Publisher’s Note

All claims expressed in this article are solely those of the authors and do not necessarily represent those of their affiliated organizations, or those of the publisher, the editors and the reviewers. Any product that may be evaluated in this article, or claim that may be made by its manufacturer, is not guaranteed or endorsed by the publisher.
